# Functional Identification of *Px-fringe* and *Px-engrailed* Genes under Heat Stress in Chlorpyrifos-Resistant and -Susceptible *Plutela xylostella* (Lepidoptera: Plutellidae)

**DOI:** 10.3390/insects11050287

**Published:** 2020-05-07

**Authors:** Yu Wang, Jingnan Wang, Xiaofeng Xia, Gang Wu

**Affiliations:** 1 Key Laboratory of Biopesticide and Chemical Biology (Ministry of Education), Fujian Agriculture and Forestry University, Fuzhou 350002, China; W1823301539@outlook.com (Y.W.); wjn5663@163.com (J.W.); 2 State Key Laboratory of Ecological Pest Control for Fujian and Taiwan Crops, Institute of Applied Ecology, Fujian Agriculture and Forestry University, Fuzhou 350002, China

**Keywords:** *fringe*, *engrailed*, resistant and susceptible *Plutella xylostella*, heat stress

## Abstract

In our previous research, the fitness cost of resistance of the diamondback moth (DBM), *Plutella xylostella* found in insecticide-resistant DBM (Rc-DBM) under heat stress was based on heavier damage to wing veins when compared to insecticide-susceptible DBM (Sm-DBM). To investigate the molecular mechanism of the damage to the veins between Rc- and Sm-DBM, the full-length sequences of two related genes involved in the development of wing veins, f*ringe* (*Px*-*fng*) and *engrailed* (*Px*-*en*) of DBM were cloned, and the mRNA expressions of both *Px-fng* and *Px-en* were studied. The *Px-fng* and *Px-en* cDNA contained 1038 bp and 1152 bp of open reading frames (ORFs), respectively, which encoded a putative protein comprising 345 and 383 amino acids with a calculated molecular weight of 39.59 kDa and 42.69 kDa. Significantly down regulated expressions of *Px-fng* and *Px-en* under heat stress were found in pupae and adults of Rc-DBM compared to Sm-DBM, and a result of higher damage to wing veins in Rc-DBM under heat stress. Based on RNAi experiments, significant inhibitions on expressions of *Px-fng* and *Px-en* in both Sm-DBM and Rc-DBM were found when the pupae were infected by dsFng or dsEn. Corresponding to these, infections of dsFng or dsEn resulted in significant decrease of eclosion rate and increase malformation rate of DBM. Our results suggest that the higher damage of wing veins in DBM might be related to the heavier inhibitions of *Px-fng* and *Px-en* expression, and the *Px-fng* and *Px-en* are involved in the development of wings and veins.

## 1. Introduction

Wings, as crucial organs for insects to resist survival stress and cope with damage, are comprised of axilla, veins, membrane, fold lines, flexion lines, and surface structures [1]. The veins serve as the skeleton for the wings and are the primary supporting structure composed of nerves, hemolymphs, and trachea [1].The development of wings is composed of three cascade signaling pathways: proximal–distal (P–D); dorsal–ventral (D–V); and anterior–posterior (A–P) [2]. The relevant genes in the three cascade signaling pathways such as *engrailed* (en), *apterous* (ap), *arista-less*, etc. determine the differentiation of the wing disc [3,4].It has also been reported that temperature affects the development of insect wings, for example, the sizes and shapes of wings are affected by temperature in *Drosophila* [5] and *P. xylostella* [6].

The gene regulatory pathway in insect wing vein development has mainly been carried out in *Drosophila* [2,7,8,9]. *Engrailed* is a nuclear regulatory protein that plays an essential role in wing morphogenesis [10] and has dual distinct functions in the development of the wing disc [11].It is known that the *engrailed* gene is correlated with the posterior cell compartment. Furthermore, *engrailed* selective expression determines the differentiation of the anterior–posterior (A–P) boundary during the whole wing development [10,12,13]. *Engrailed* interacts with some other proteins and could be post-translationally manipulated to increase the level of genetic diversity [14,15]. Moreover, for some insects, *Engrailed*-family genes function as a switch to regulate the wing color patterns [16]. *Fringe* was first discovered because it plays a critical role in locating Notch activation of special boundary cells along the dorsal ventral septal boundary of the wings [17].It is well known that *fringe* is a glycosyltransferase, which is located in the Golgi and plays a part in the Notch [18].Fringe-modulated deployment of Notch signaling is important to the boundary formation of wings [19].Fringe protein encodes a boundary-specific cellular signaling molecule that is responsible for the interaction between dorsal–abdominal cells during wing development [17]. It has been reported that the loss of *fringe* expression could lead to an abnormal development of wings boundary, and can even result in wing loss [17,20].

In addition to the studies on *Drosophila*, there have also been some studies on the development of venation in other insects in recent years. A pupa-specific expression of chitin synthase A 2b (CHSA-2b) in *Bombyx mori* was identified. This protein was expressed in the wing in mid-pupal stage and regulates the wing development [21,22]. The hormone 20-hydroxyecdysone (20E) regulates the expression of CHSA-2b to mediate the wing formation [21,22]. β-N-acetylglucosaminidases (NAGs) is a group of enzymes that degrade chitino ligosaccharides, which is important for insect development and metamorphosis in *Lasioderma serricorne*. Knockdown of β-N-acetylglucosaminidase 2 decreased many other wing-development-related genes, thus affecting the molting and wing development [23]. In the brown planthopper (BPH) *Nilaparvata lugens*, knockdown of the decapentaplegic (dpp) gene causes different phenotypes in the long-winged (LW) and short-winged (SW) morphs, but both cause the absence of wing veins and affect the wing morph transformation [24]. Earlier studies have also revealed that dpp is closely related to the development of the wings and veins of *Drosophila* [25]. The directional transport of dpp may generate diversified patterns of insect wing vein [26]. Similarly, in the sawfly *Athalia rosae*, the decapentaplegic (dpp)/bone morphogenetic protein (BMP) signal pathway plays an important role in the development of the wing vein. The dpp transport system and the spatial transcription factors of the BMP significantly affect the development of wings and veins [27,28].

DBM is a global economic pest that feeds on cruciferous crops. Meanwhile, DBM has developed resistance to several insecticides [29,30,31,32]. In our previous study, heat stress can affect the fitness cost of resistant DBM. Compared to chlorpyrifos-susceptible DBM, chlorpyrifos-resistant DBM has a significantly lower fertility, thermal tolerance, heat shock protein expression, but higher germ and ovary cell damage, and an upregulating expression of mitochondrial apoptosis-related genes (*caspase-9*, *Apaf-1b, cytochrome c*) [6,33,34,35]. The results indicated that Rc-DBM showed significant fitness cost caused by insecticide resistance. Under insecticide screening, the ability of insects to resist environmental stress decreased to a certain extent, which was the fitness cost. The fitness cost refers to the ability of insects to adapt to survive in the natural environment and pass this trait to the next generation through genes, which generally include fecundity and viability [36]. We also found that nine genes in chlorpyrifos-resistant DBM, which were thought to be involved in wing development in *Drosophila* [2], had a significant downregulated expression under heat stress when compared to chlorpyrifos-susceptible DBM [37]. Although significantly down regulated expressions of the nine genes under heat stress [37] and vein damage were found under heat stress in Rc-DBM [6], it was unclear if the nine genes were involved in the wing development of DBM. As *fringe (fng)* and *engrailed* (en) are thought to be wing-development-related genes [2], in this study, *fringe (Px-fng)* and *engrailed* (*Px-en*) were identified, and the mRNA dynamic expressions of *Px-fng* and *Px-en* were investigated in chlorpyrifos-resistant and -susceptible DBM under heat stress. In addition, the function of the two genes in the wing development of DBM was confirmed by using RNA interference (RNAi).

## 2. Materials and Methods 

### 2.1. Source of Insect

Chlorpyrifos-resistant (Rc) and -susceptible (Sm) strains of DBM were kept in our laboratory for a long time. The information about the two strains of DBM was described in detail in our previous study [34].

### 2.2. Cloning of Fringe (Px-fng) and Engrailed (Px-en) Genes

Sample: *fringe* and *engrailed* gene in DBM were named as *Px-fng* and *Px-en* in this study, respectively. For cloning *fng* and *en,* fourth instar larvae from Sm-DBM were collected and reared at 25 °C and 80% relative humidity (RH) with a 16:8 h light/dark (L:D) photoperiod in an environmental chamber. The F1 progenies of the pupae and adults were used for the total RNA extraction.

### 2.3. Amplification of the Initial Fragments of Fringe (Px-fng) and Engrailed (Px-en)

The MiniBEST Universal RNA Extraction Kit (TaKaRa Bio Inc., Dalian, China) was used to extract total RNA from different samples. We compared the *fng* and *en* sequences in NCBI with other insects to find the conservative regions of cDNA and tried to design primers using Primer Premier 5.0 (Table 1).The polymerase chain reaction (PCR) system was 50 μL, the optimum reaction system of PCR wasTaKaRaExTaq0.25 μL, 10× ExTaq Buffer 5.0 μL, dNTP Mixture4.0 μL, Template 1.0 μL, Primer 1 1.0 μL, Primer 21.0 μL, and ddH_2_O 37.75 μL. We used a 2720 Thermal Cycle for this PCR reaction. The PCR condition was: 95 °C denaturation for 3 min, followed by 35 cycles of 94 °C 30 s, 59.9 °C 1 min for *Px-fng*, or 57.8 °C 1 min for *Px-en*, then extension at 72 °C for 1 min, and 72 °C 10 min for the final reaction. PCR products were detected by electrophoresis on 1% agarose gel. The specific DNA fragments were purified with the MiniBEST agarose gel DNA Extraction Kit. Then, it was connected to a pMD20-T vector and finally transfected into competent cells. The bacterial solution was sent to be sequenced by Fuzhou Boshang Biotechnology Co. Ltd.

### 2.4. Rapid Amplification of cDNA Ends (RACE) of Fringe (Px-fng) and Engrailed (Px-en)

The SMARTer^®^ RACE 5′/3′ Kit (Clontech Laboratories, Inc., Mountain View, USA) was used to clone the 3′- and 5′-cDNA sequences of *Px-fng* and *Px-en*. The total volume of the PCR system was 50 μL, the optimum reaction system of PCR was Ex-Taq (5 U/μL) 0.25 μL, dNTP-Mix (2.5 mM) 4.0 μL, 5′-or 3′-RACE-Ready cDNA 3.0 μL, 5′ or 3′ GSP (10 μm) 1.0 μL, 10× UPM 1.0 μL, 10× Ex-Taq Buffer 5.0 μL, and ddH_2_O 35.75 μL, based on the intermediate sequences we have previously cloned (Table 2). The PCR condition was: 94 °C 3 min, 94 °C 30 s, 68 °C 30 s, 72 °C 3 min, 25 cycles, 72 °C 7min.

### 2.5. Amplification of open Reading Frames (ORFs)

The previous three sequencing results were spliced to obtain the full cDNA sequences. Using the NCBI online ORF Finder (http://www.ncbi.nlm.nih.gov/gorf/gorf.html) to identify the ORFs. In order to verify the accuracy of full-length gene splicing and eliminate the problem of inaccurate tail sequence in the process of sequence splicing, specific primers were designed at both ends to amplify the ORF sequences, respectively (Table 1). PCR conditions was: The total volume of the PCR system was the same as the amplification of the initial fragments, 94 °C 5 min, followed by 35 cycles at 94 °C 30 s, annealing at 52 °C 30 s for *Px-fng*, 55 °C 30 s for *Px-en*, extension at 72 °C 2 min, with final reaction of 72 °C 8 min.

### 2.6. Real-Time Quantitative Polymerase Chain Reaction (qPCR)

#### 2.6.1. Temperature Shock

In this experiment, adults or pupae of Rc- or Sm-DBM were treated with heat stress. The high temperature treatment mimicked the field micro-climate temperature in Fuzhou in summer [6]. The temperature treatment was divided into four groups based on our previous study [6], the control group was treated at 25 °C for different times, and the corresponding experimental group was treated at different temperatures: Group (1): 25 °C for 1 h as the control group, 44 °C for 1 h as the treatment group; Group (2): 25 °C for 4 h as the control group, 42 °C for 4 h and 8 has the treatment group; Group (3): 25 °C for 8 has the control group,40 °C for 8 h and 16 has the treatment group; and Group (4): 25 °C for 48 has the control group,38 °C for 48 has the treatment group. Before the total RNA extraction, the high-temperature treated DBM should recover at 25 °C for 1 h.

#### 2.6.2. Determination of mRNA Expression

We used the SYBR^®^ GREEN (Takara, Dalian, China) fluorescent method in the 7500 Fast RealTime PCR System to complete the determination of *Px*-*fng* and *Px*-*en* expression quantity. All the experimental samples under went three biological repeats (with ten insect individuals for each replication) and three technical repeats for each temperature treatment. Primers for quantitative fluorescence PCR were designed by the primer premier 5.0 (Table 2). Meanwhile, β-actin and Elongation factor (Ef) were used as the house-keeping genes. The total volume of the PCR system was 20 μL, the optimum reaction system of PCR was 5× PrimeScript Buffer 4.0 μL, PrimeScript RT Enzyme Mix I 1.0 μL, Oligo dT Primer (50 μM) 1.0 μL, Random 6 mers (100 μM) 1.0 μL, Total RNase Free ddH_2_O up to 13.0 μL. PCR conditions was: 95 °C 30 s, 95 °C 5 s, 60 °C 34 s, 40 cycles in total. The experimental data were obtained and analyzed using the 2^−^^△△Ct^ method. 

### 2.7. Functional Analysis of Px-fng and Px-en on Deformities and Damage in DBM Wings by RNAi

To explore the effects of *Px-fng* and *Px-en* in DBM wing development, RNAi was used to silence the expression of *Px-fng* and *Px-en*. Based on the entire coding sequence of *Px-fng* and *Px-en,* the double stranded RNA (dsRNA) and qPCR primers were designed (Table 3). Meanwhile, the dsGFP was synthesized as a negative control (Table 3) [38]. As vein differentiation in a wing disc occurs at the pupae stage, pupae were used for the RNAi experiments. The pupae were divided into four groups. In each group, seventy pupae were injected with 69 nL and 138 nL (2 μg/μL), dsGFP, dsFng, and dsEn using Nanoliter 2000/B203XVB (WPI) at 25 °C for 4 h, 8 h, 16 h, and 48 h, respectively. According to the survival rate and the effect of RNAi, the injection amount of each pupa was determined to be 138 nl dsRNA in each insect (Table 4). The hind wings of emerged adults were collected and the wing scales were gently rinsed out with 70% ethanol to make the veins appear more clearly [6]. Then, they were observed by zoom stereoscopic microscope (Nicon SMZ18, Tokyo, Japan). The wing deformity was assumed to occur when different sizes and shapes of the wings were found compared to the normal wings. The degree of wing damage was determined by the two hind wings of each adult, because damage may occur on one or both hind wings of an adult. In addition, since damage may occur on one or both hind wings of any adult, vein damage is considered to occur when the radinus posterior (Rp) vein disappears [6]. Three biological replications were performed with 70 individual insects for each replication in the observations on the effects of RNAi on the wing morphology of DBM. Six individuals among them were used in the expression analysis of mRNA.

### 2.8. Multiple Sequence Alignment and Phylogenetic Analysis

For the multiple sequence alignment of *Px-fng* and *Px-En*, the deduced amino acid sequences of *Px-fng*/*Px-En*, and the related sequences (*Fringe*/*Engrailed*) known in other species were aligned by DNAMAN V6, respectively. For the phylogenetic analysis, the amino acid sequences of *Fringe*/*Engrailed* of other insects were downloaded from the NCBI database, and their homologous sequences, brainiac and invected, were downloaded, respectively. The ClustalW program in MEGA6.0 [39] was used for multiple sequence alignment, the phylogenetic trees constructed by the neighbor-joining method were verified by bootstrap.

### 2.9. Statistical Analysis

The data of the effects of heat stress on the expression of *Px-fng* and *Px-en* in DBM, the survival rate and gene expression level of the DBM when injected with dsRNA were analyzed by the Statistical Product and Service Solutions (SPSS21, IBM, Armonk, USA) software. Duncan’s test was used for the analysis of the difference among the multiple group of samples. 

## 3. Results

### 3.1. Cloning and Sequences Analysis of Px-fng and Px-en

**Figure 1:**
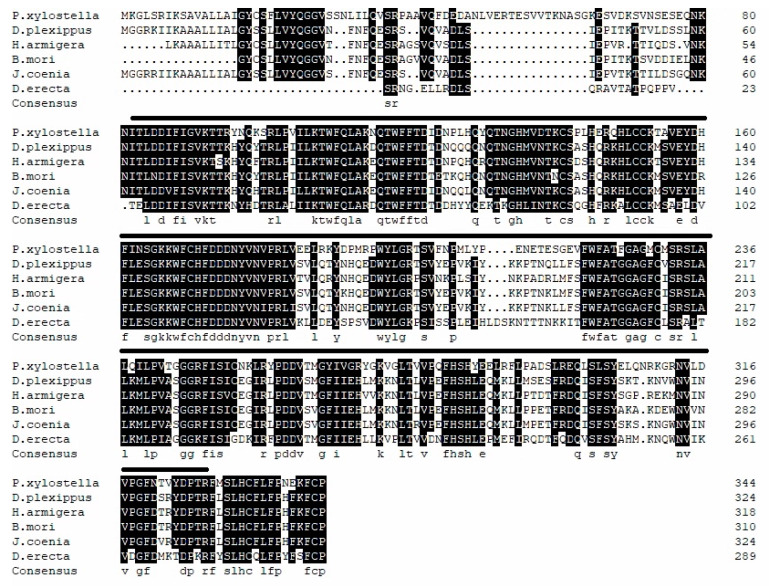
Multiple alignments of Px-fng. The amino acids labeled below represented identical residues in all the species. Lines above the sequences defined the conserved domain of Fringe. The GenBank number of the sequences were: *P. xylostella* (MK530647.1), *D. plexippus* (OWR44193.1), *H. armigera* (XP_021183549.1), *B. mori* (NP_001116813.1), *J. coenia* (AAO38754.1), and *D. erecta* (XP_001973555.1).

**Figure 2:**
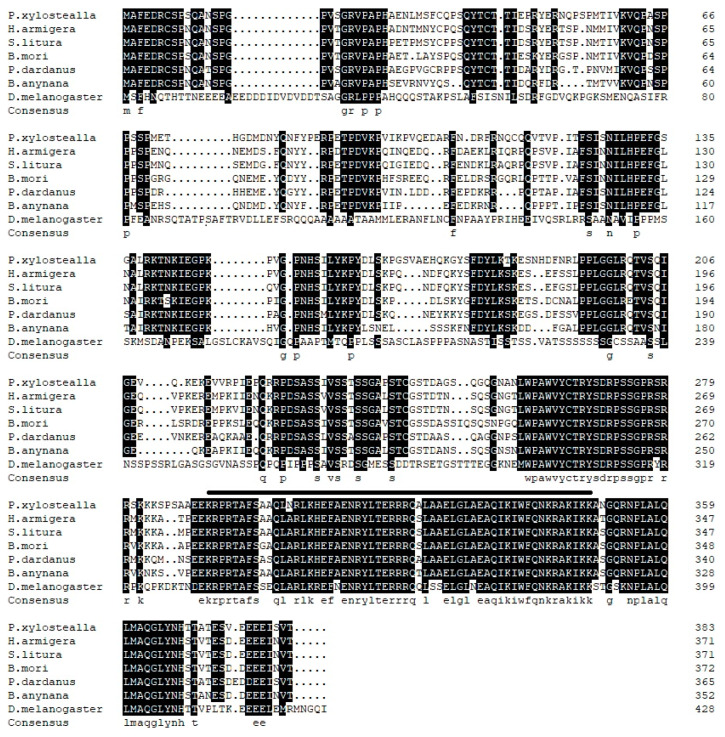
Multiple alignments of Px-En. The amino acids labeled below represented identical residues in all the species. Lines above the sequences defined the conserved domain of Engrailed. The GenBank number of the sequences were: *P. xylostella* (MK530646.1), *H. armigera* (XP_021180829.1), *S. litura* (XP_022817253.1), *B. mori* (NP_001037550.2), *P. dardanus* (CAX36786.1), *B. anynana* (XP_023948032.1), and *D. melanogaster* (BAN82731.1).

Based on the 3′- and 5′-RACE amplification fragments and first cDNA fragments, the full-length cDNA of Px-*fng* and Px-*en* were cloned, with the NCBI GenBank numbers MK530647 and MK530646, respectively. The full-length of *Px-fng* was 2140 bp and *Px*-*en* was 2361 bp, and the open reading frame (ORF) sequences were 1038 bp for *Px*-*fng* and 1152 bp for *Px*-*en*, respectively. The *Px*-*fng* encodes 345 amino acids. Moreover, it also has a transmembrane helix region(7aa–26aa) and a *Fringe* functional domain (80 aa–328 aa). ExPASy analysis deduced a molecular weight of 39.59 KDa and predicted an isoelectric point of 8.01. The *Px*-*en* encodes 383 amino acids. In addition, it contains a HOX domain from 297 aa to 359 aa. ExPASy analysis deduced a molecular weight of 42.69 KDa and predicted an isoelectric point of 9.31. According to the Blast results, the obtained *Px-fng* and *Px-en*amino acid sequences of DBM were consistent with that of other insects, for *Px-fng*, 57% identity with *Danaus plexippus* (OWR44193.1), 59% with *Helicoverpa armigera* (XP_02 11 8354 9.1), 54% with *B. mori* (NP_001116813.1), 57% with *Junonia coenia* (AAO38754.1), and 42% with *Drosophila erecta* (XP_001973555.1) (Figure 1); for *Px*-*en*, 76% with *H. armigera* (XP_021180829.1), 77% with *Spodopteralitura* (XP_022817253.1), 73% with *B. mori* (NP_001037550.2), 73% with *Papilio Dardanus* (CAX36786.1), 72% with *Bicyclusanynana* (XP_023948032.1), and 32% with *Drosophila melanogaster* (BAN82731.1) (Figure 2). Phylogenetic analysis showed that Px-fng and Px-en were clustered together with the fringe and engrailed proteins in other Lepidoptera insects, and formed independent populations in the phylogenetic trees with their homologs, brainiac and infected, respectively (Figures S1 and S2).

### 3.2. Expressions of Px-fng and Px-en under Heat Stresses in Pupae and Adults

**Figure 3:**
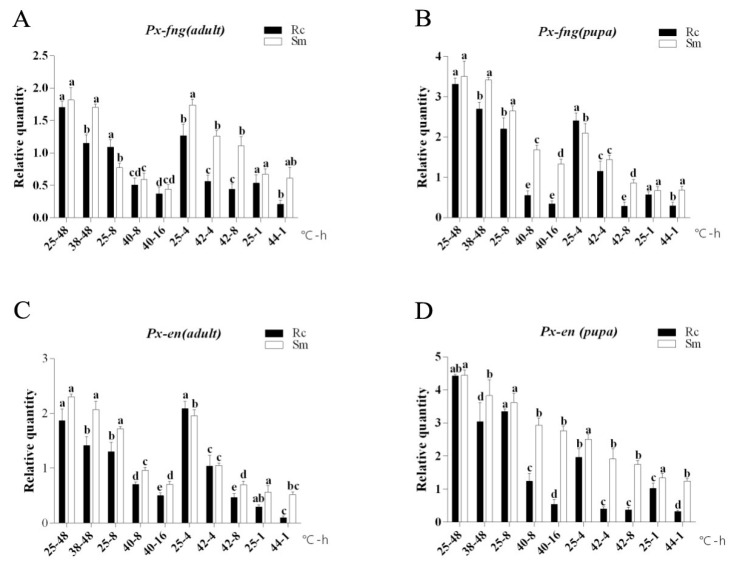
Different heat stress on the expression of *Px*-*fng* and *Px*-*en* in DBM. Rc: insecticide-resistant DBM, Sm: insecticide-susceptible DBM. Abscissa: temperature (°C)-treated time (h). Ordinate: the relative transcription expression (Mean ± SE) of *Px*-*fng* and *Px*-*en*, respectively. (**A**): Effects of heat stress on the expression of *Px*-*fng* in adult DBM. (**B**): Effects of heat stress on the expression of *Px*-*fng* in pupa DBM. (**C**): Effects of heat stress on the expression of *Px*-*en* in adult DBM. (**D**): Effects of heat stress on the expression of *Px*-*en* in pupa DBM. Duncan’s test was used for the analysis of the difference between different groups of samples. According to the four groups of experiments designed above (different temperature treatment), each group was analyzed separately, and then these data were integrated into one figure. Therefore, different lowercase letters in the figures represent the statistically significant difference (Duncan’s test, *p* ≤ 0.05) between each sample in the corresponding groups, and the same letter means that there is no significant difference.

The mRNA expression of *Px-fng* and *Px-en* in pupae or adults of Rc- and Sm-DBM under heat stress showed that: the basal levels (at 25 °C) of *Px-fng* and *Px-en* expression were high in adults or pupae of both Rc- and Sm-DBM, however, there was no significant difference between Rc- and Sm-DBM. In the group of 25 °C for 48 h and 38 °C for 48 h, the expression of *Px-fng* and *Px-en* in adults and pupae were significantly downregulated under heat stress (38 °C) than the basal level (25 °C) in both Rc- and Sm-DBM, but the downregulation degree of Rc-DBM was more obvious. The same phenomenon under heat stress expression were also be found in 40 °C, 8 h; 40 °C, 16 h; 42 °C, 4 h; 42 °C, 8 h and 44 °C, 1 h when compared with 25 °C (Figure 3).

### 3.3. Px-fng and Px-en Expression in Response to dsRNA in DBM Wings

**Figure 4:**
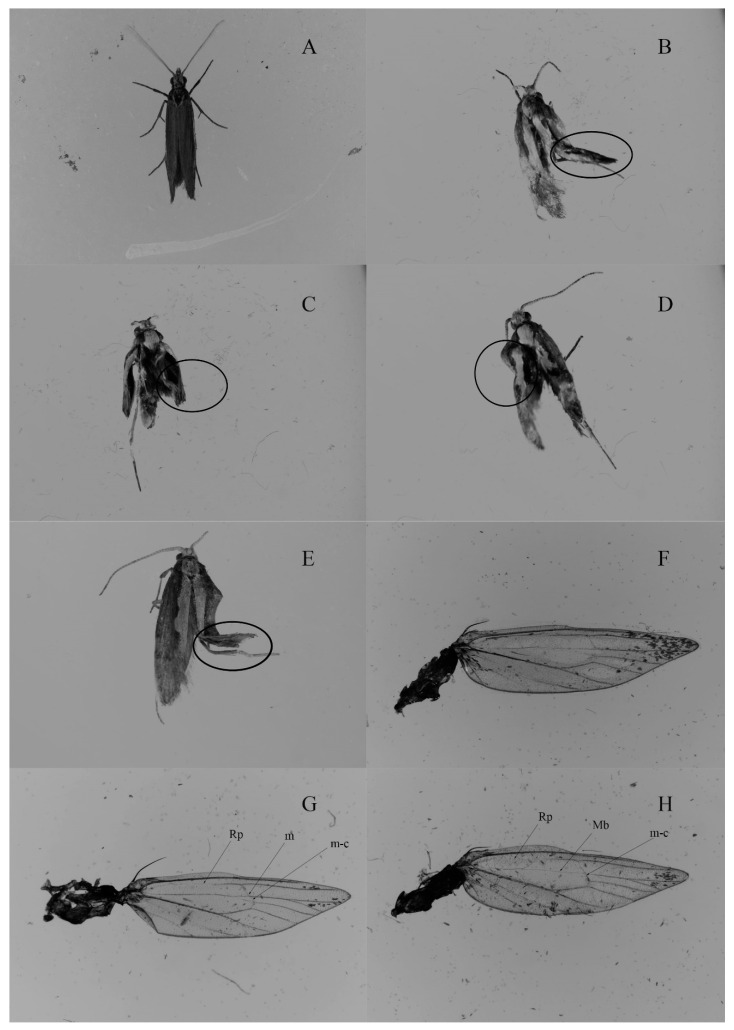
Normal and wing deformity in DBM adults. (**A**) Normal adult. (**B**,**C**) Wings with phenotypic changes after treatment with dsFng. (**D**,**E**) Wings with phenotypic changes after treatment with dsEn. (**F**) Wing with normal veins. (**G**,**H**) Wings with the vein missing or weakening (indicated by an arrow) after the injection of dsFng and dsEn, respectively, Rp: Radinus posterior, m: Medial crossvein, m-c: Medialcubitus crossvein, Mb: Medial bar.

**Figure 5:**
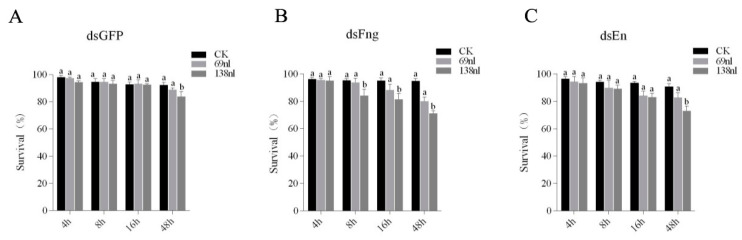
The survival (%) of the pupae of DBM when injected with different doses of dsFng, dsEn, and dsGFP. A total of 138 nl dsRNA (2 μg/μL) was selected for RNAi as it can achieve the best interference effect while maintaining a sufficient survival rate (more than 70 percent). (**A**): The survival (%) of the pupae of DBM when injected with different doses of dsGFP, (**B**): The survival (%) of the pupae of DBM when injected with different doses of dsFng, (**C**): The survival (%) of the pupae of DBM when injected with different doses of dsEn. Data in the figures were represented by mean ± SE, and the Duncan’s test was used for the analysis of the difference in each treatment. The different lowercase letters indicate significant differences (Duncan’s test, *p* ≤ 0.05) in each sample under different treatments at each time point, respectively.

**Figure 6:**
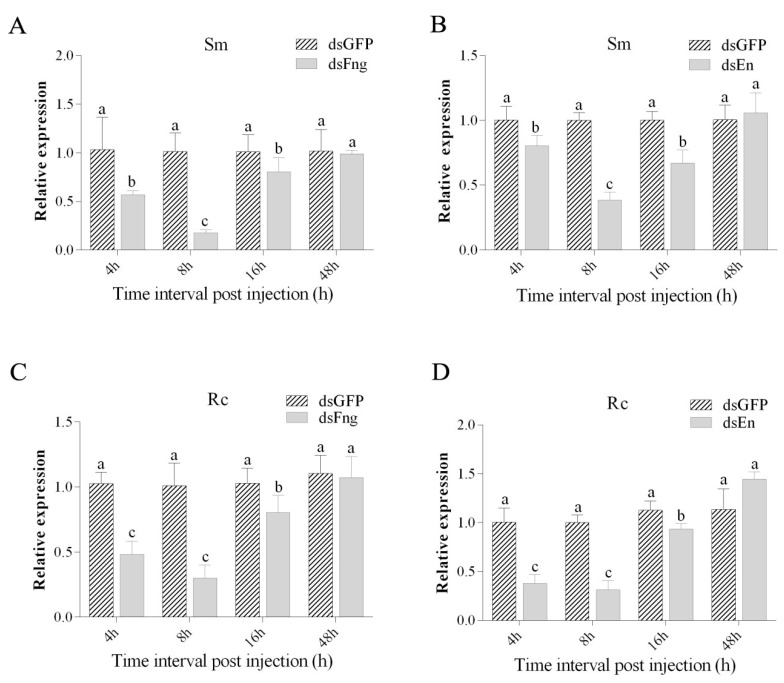
Comparison of *Px-fng* and *Px-en* expression levels in Rc-and Sm-pupae by dsFng or dsEn at different times after treatment. (**A**): *Px-fng* expression in Sm-pupae by dsFng treatment, (**B**): *Px-en* expression in Sm-pupae by dsEn treatment, (**C**): *Px-fng* expression in Rc-pupae by dsFng treatment, (**D**): *Px-en* expression in Rc-pupae by dsEn treatment. Duncan’s test was used for the analysis of the difference among different groups of samples. Different lowercase letters indicate significant differences in each sample (both Rc and Sm) at different time point after RNA interference (Duncan’s test, *p* ≤ 0.05). The x-axis indicates the time interval post injection.

In order to determine the most suitable doses of dsRNA and the observation time of interference, different doses of dsRNA were used to inject the DBM, and the results suggest that 138 nl dsRNA can achieve the best interference effect while maintaining a sufficient survival rate (Table 4, Figure 4 and Figure 5). After injection of dsFng or dsEn, among 210 pupae, the emergency rate of pupae was 72% or 73%, respectively, and the wing deformity rate was 37.75% or 40.52%, respectively. However, after injection of dsGFP and dsH_2_O, the emergency rate of pupae was as high as 94% or 91%, respectively, and no wing damage was found (Table 4, Figure 4 and Figure 5). Wing damage usually manifests itself as a loss of one or more veins of the hind wing. After the injection of dsFng or dsEn into the pupae, in addition to the Rp vein loss, Mb, m and m-c were also weakened, although not as obvious as Rp [6]. Compared to the control group (injected with dsGFP), the expression of *Px-fng* and *Px-en* was significantly suppressed in both Sm-and Rc-pupae at 4h, 8h, and 16h after being injected with dsFng and dsEn (Figure 6). However, no significant inhibitions on the expression of *Px-fng* and *Px-en* were found in Sm- or Rc-pupae 48h after being injected with dsFng and dsEn (Figure 6).

## 4. Discussion

In the present study, two cDNA sequences from the DBM were identified and characterized. From the amino acid analysis, we determined that Px-fng and Px-en proteins of DBM had a high conserved region compared to other species. Furthermore, the phylogenetic analysis confirmed that both the Px-fng and Px-en with other Lepidopteran insects had the closest genetic relationship. The above analysis also supports the fact that the cloned sequences were our target genes.

According to previous research results, among the wing-development-related genes, the mRNA expression of those genes was significantly downregulated in Rc-DBM compared to Sm-DBM under heat stress [37]. In our present study, for the pupae or adults, the expression of both *Px-fng* and *Px-en* declined under heat stress. The Rc-strain showed more significant downregulation in mRNA expression than Sm under heat stress. It is speculated that, to some extent, this phenomenon is related to the fact that the damage degree of Rc-DBM wing veins are higher than that of Sm-DBM under thermal stress [6].

Temperature is an important climatic factor, which can affect the ecological and evolutionary processes of organisms. In recent years, with global warming, the effect of temperature on insect fitness has become the focus of research. Heat stress is also deleterious to fitness costs [40]. Particularly in insects, there have been many reports, for example, *Xestia c-nigrum* (Lepidoptera: noctuidae) showed delayed development [41] and *Trichogrammacarverae* reduced its parasitic rate [42]. In our previous study, different temperature treatments can affect the fitness cost of Rc- and Sm-DBM. The Sm-strain is more suitable for high temperatures than the Rc-strain [6]. In this study, *Px-fng* and *Px-en*in Rc-DBM under heat stress had significantly downregulated expression compared to Sm-pupae. As the *Px-fng* and *Px*-*en* expressions were inhibited after an injection of dsFng and dsEn, a deformity of veins occurred (Figure 4, Figure 5 and Figure 6). It was speculated that these two genes might be involved in the developments of vines. In addition, it should be noted that, in our present study, *Px-fng* and *Px-en* were confirmed to be involved in the development of the wings in DBM based on dsRNA. However, aside from malformation and vein damage, wide-spread phenotypes (Figure 4) such as the effects on other tissues including the body, antenna, and leg, were observed in RNAi treatments, which implicated that *Px-fng* and *Px-en* might be involved in the development of many tissues throughout the body of DBM. In *Drosophila*, both *engrailed* and *fringe* were confirmed to be expressed in many tissues throughout the body [14,18]. In our experiment, we injected dsRNA into the pupae, which showed non-specificity to the wing, but led to many other tissue malformations. Nevertheless, our study established a relationship between these two genes (*Px-fng* and *Px-en*) and the development of the wings of DBM for the first time. However, their specific regulatory mechanisms still need to be further studied in the future such as studying the specific expression regulation of their wings to find out their effects on wing development.

In this study, only Rc-DBM was used to study the effects of RNA interference on wing deformity and wing damage (Table 4 and Figure 4), although the comparisons of the effects of RNA interference on *Px-fng* and *Px-en* expression between Rc- and Sm-DBM were carried out (Figure 6). However, compared to Sm-DBM, higher vein damage in Rc-DBM under heat stress was confirmed in our previous study [6]. In the present study, *Px-fng* and *Px-en* were first confirmed to be involved in the development of wings and veins based on RNAi experiments. Our results also confirmed that the inhibition of heat stress on the expression of two genes of Rc is significantly greater than Sm. Thus, we proposed that the more severe damage of Rc venation compared to Sm under heat stress may be related to the more serious inhibition of *Px-fng* and *Px-en*. However, it is important to pay attention to the absence of the vein after RNAi, which was mainly concentrated in Rp in this study. In addition, Mb, m, and m-c were also weakened, but not as obviously as Rp. In our previous studies, the absence of the vein caused by heat stress occurred mainly in Mb, m, m-c, and r-m [6], which indicated that the use of RNAi and a heat stress treatment would both lead to the absence of the vein, but the results were not the same, suggesting that there is something different between the mechanisms of heat stress and RNAi on the development of a vein. Heat stress leads to changes in the expression of multiple genes including *Px-fng* and *Px-en*, but *Px-fng* and *Px-en* may be the key genes in the developmental path of DBM venation. The inhibition of these two genes by RNAi may also affect the expression of multiple genes related to this path. Therefore, RNAi and heat stress lead to the deletion of venation, which may be influenced by both common genes and their own specific gene expression. As for RNAi and heat stress, it is necessary to further study the mechanism in the absence of the vein, respectively.

## 5. Conclusions

Our results suggest that heat stress downregulated the wing development genes *Px-fng* and *Px-en*, thus resulting in damage to wing veins and fitness cost in the Rc-DBM strains. The fitness cost in Rc-DBM in the previous studies [6,33,34,35] and the current study might be related to the alleles ace1R which mediates the resistance of many organophosphorus pesticides [34,35]. However, this hypothesis still needs further verification. The study of the cost of resistance fitness of DBM from the perspective of a morphological difference under heat stress is an innovative topic in this field. It will be helpful to clarify the sharp decline of the resistance level of DBM during the high temperature period of summer in the field, which will help to further study the physiological response, heredity, and the evolution of insects under environmental stress. In the case of global warming, our research on the influence of temperature on the resistance level of DBM has important guiding significance for the control of DBM in the field.

## Figures and Tables

**Table 1:** Primers used for cloning *fringe* (*Px-fng*) and *engrailed* (*Px-en*) genes of the diamondback moth (DBM).

Names of Primers		Sequences of Primers (5′–3′)	Tm °C	Isolated Gene
**For initial fragment(s)**				
en-1F		5′ CCGTGATAAACCCAGTCCAA 3′	57.8	Px-en-1-1
en-1R		5′ GCTTTCAGTGGCTGTCGTGT 3′	59.9	
fng-1F		5′ TTCCTGGTTTATCAAGGTGG 3′	55.8	Px-fng-2-1
fng-1R		5′ AATACAGTCATGTCGCTCAA 3′	53.7	
**For RACE**				
en-3-1		5′ TTGGAGGGTTGAGGCAGACG 3′	61.9	Px-en-3-1
en-3-2		5′ CCTGTTTGCGTTATTGTCACG 3′	61.9	
en-5-1		5′ TGCCTCAACCCTCCAAGCGG 3′	64	Px-en-5-1
en-5-2		5′ GGTCGTATGGCTTGTAAAGAAT 3′	56.3	
fng-3-1		5′ GCAAAACAGAAAGGGACGGA 3′	57.8	Px-fng-3-1
fng-3-2		5′ TGAGACGGAAAGCGGCGAGG 3′	64	
fng-5-1		5′ TGAAGCGGGTTGTCTATGTCCGTG 3′	63.7	Px-fng-5-1
fng-5-2		5′ TGATACTGATGAAGCGGGTTGTC 3′	60.2	
UPM	L*	5′CTAATACGACTCACTATAGGGCAAGCAGTGGTATCAACGCAGAGT3′		
	S*	5′CTAATACGACTCACTATAGGGC3′		
**For ORF**				
fng-C-1F		5′ ATGAAAGGGCTAAGCAGAAT 3′	53.7	
fng-C-1R		5′ ACAAAACAGGGAATCAACAT 3′	51.7	
en-C-1F		5′ CCACGGCATACATTATCATC 3′	55.8	
en-C-1R		5′ GCCTCTACCCATTACAACAA 3′	55.8	

* The primer sequences were as described in the SMARTer^®^ RACE 5′/3′ Kit.

**Table 2:** Primers used for qPCR identification of *Px*-*fng* and *Px*-*en* in DBM.

Primers	Sequences of Primers (5′–3′)	Gene Names	Product Size(bp)
β-actin-F	5′CCGAGAGAGAAATCGTGCGT 3′	β-actin	110
β-actin-R	5′GTAGGACTTCTCGAGCGAGC 3′		
Ef-F	5′AGATGCACCACGAAGCTCTC 3′	Px-ef	118
Ef-R	5′TTGTTCTTGGAGTCTCCGGC 3′		
Px-fng-q-F	5′CGGACATAGACAACCCGCTT 3′	Px-fng	135
Px-fng-q-R	5′TCCCGCTGTTGATGAAGTGG 3′		
Px-en-q-F	5′CGCGGAGAATCTCATGAGCT 3′	Px-en	112
Px-en-q-R	5′TGGCTGGACCTTCACAATGG 3′		

**Table 3:** Primer sequences for dsRNA amplification and qPCR.

Primers for PCR	Sequences of Primers (5′–3′)
**Primers for dsRNA Amplification**	
dsFng-F	5′TAATACGACTCACTATAGGGCTTGGCATGAAAGGGCTAAG3′
dsFng-R	5′TAATACGACTCACTATAGGGTGAAGTGGTCGTACTCGACG3′
dsEn-F	5′TAATACGACTCACTATAGGGCCGAGGTATGAGAGGAACCA3′
dsEn-R	5′TAATACGACTCACTATAGGGTTCTGAACCTCCCCAATCTG3′
**Primers for qPCR**	
RT-qPCR dsFng-F	5′GTAGGTCGCTACGGCAAAGT3′
RT-qPCR dsFng-R	5′CACGTTCCGTCCCTTTCTGT3′
RT-qPCR dsEn-F	5′ATCGATGACGCGACGATTCA3′
RT-qPCR dsEn-R	5′AACACACACAACGGCGATTG3′
dsGFP-F	5′TAATACGACTCACTATAGGGCAGTGCTTCAGCCGCTAC3′
dsGFP-R	5′TAATACGACTCACTATAGGGGTTCACCTTGATGCCGTTC3′-

**Table 4:** Statistics on the emergence and wing deformity when dsFng and dsEn were injected.

Genes Injected	Numbers Tested	Emergence (Mean ± SE) (%)	Deformity (Mean ± SE) (%)
dsFng	210	71.9 ± 2.65b	37.9 ± 2.76b
dsEn	210	72.85 ± 2.85b	40.6 ± 3.38b
dsGFP	210	94.28 ± 1.64a	0a
dsH2O	210	90.95 ± 1.25a	0a

Note: 138 nl dsFng, dsEn, dsGFP, or ddH_2_O was injected to each pupa, respectively. Different lowercase letters in each volume indicate significant differences in each treatment (Duncan’s test, *p* ≤ 0.05).

## Supplementary Materials

The following are available online at https://www.mdpi.com/2075-4450/11/5/287/s1, Figure S1: Phylogenetic tree of fringe in different species of insects, Figure S2: Phylogenetic tree of engrailed in different species of insects.
