# Supplementary material for: Functional Identification of Px-fringe and Px-engrailed Genes under Heat Stress in Chlorpyrifos-Resistant and -Susceptible Plutela xylostella (Lepidoptera: Plutellidae)
Source: Insects. 2020 May 7;11(5):287. doi: 10.3390/insects11050287 (PMC7290670; doi:10.3390/insects11050287)
Supplement: Supplementary file 1 [file insects-11-00287-s001.pdf]

Supplementary Materials

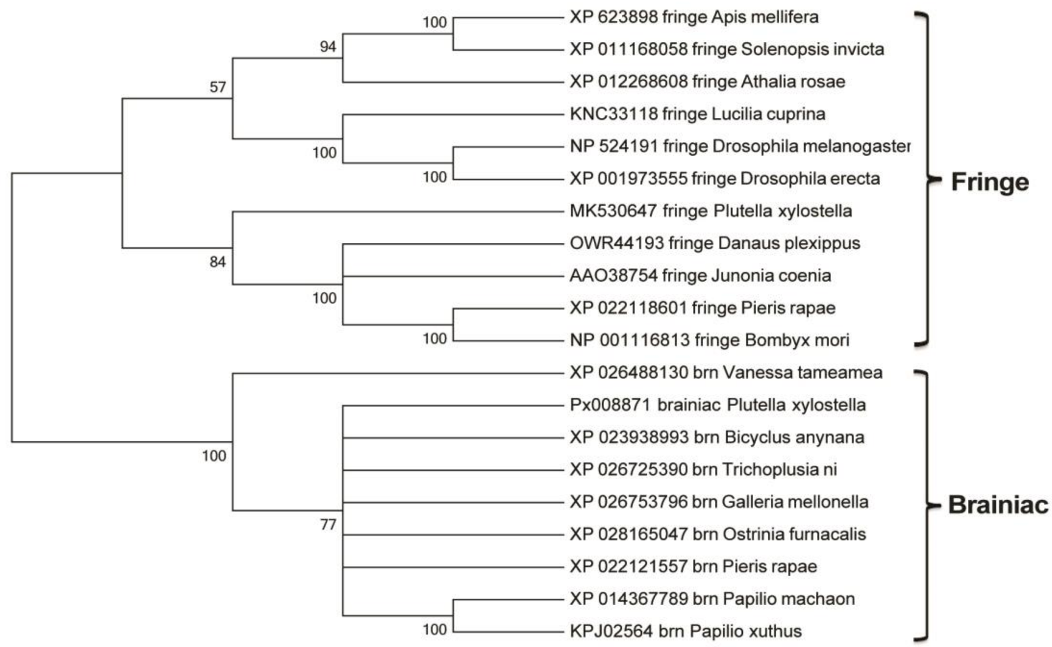

Figure S1. Phylogenetic tree of fringe in different species of insects: the fringe for *Apis mellifera* (XP_623898.4); *Solenopsis invicta* (XP_011168058.1); *Athalia rosae* (XP_012268608.1); *Lucilia cuprina* (KNC33118.1); *Drosophila melanogaster* (NP_524191.1); *Drosophila erecta* (XP_001973555.1); *Plutella xylostella* (MK530647.1); *Junonia coenia* (AAO38754.1); *Danaus plexippus* (OWR44193.1); *Pieris rapae* (XP_022118601.1); *Bombyx mori* (NP_001116813.1), and the brainiac for *Plutella xylostella-* (Px008871 in the Genome database of *P. xylostella*, http://59.79.254.1/DBM/index.php), *Galleria mellonella* (XP_026753796), *Papilio machaon* (XP_014367789), *Papilio xuthus* (KPJ02564), *Bicyclus anynana* (XP_023938993), *Trichoplusia ni* (XP_026725390), *Pieris rapae* (XP_022121557 brn), *Vanessa tameamea* (XP_026488130), *Ostrinia furnacalis* (XP_028165047), the brainiac is homolog to fringe, however, the results of phylogenetic analysis showed that the braniac and fringe were divided into two independent groups on the evolutionary tree. 1000 bootstrap was used to evaluate the phylogenetic tree, and the cut-off value for condensed tree was 50%.

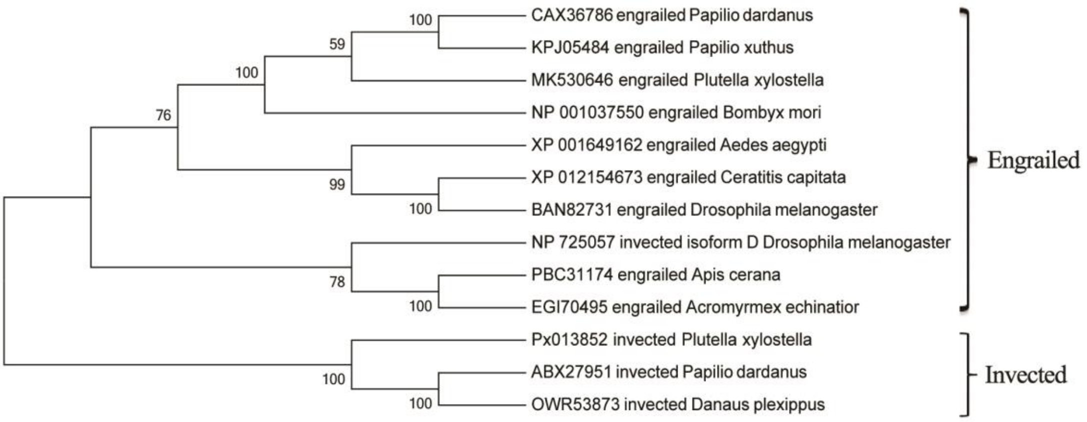

Figure S2. Phylogenetic tree of engrailed in different species of insects: the engrailed for *Drosophila melanogaster* (BAN82731.1); *Ceratitis capitata* (XP_012154673.1); *Aedes aegypti* (XP_001649162.2); *Acromyrmex echinatior* (EGI70495.1); *Apiscerana cerana* (PBC31174.1); *Plutella xylostella* (MK530646.1); *Bombyx mori* (NP_001037550.2); *Papilio dardanus* (CAX36786.1); *Papilio xuthus* (KPJ05484.1), and the invected for *Plutella xylostella* (Px01385 in the Genome database of *P. xylostella*,

http://59.79.254.1/DBM/index.php), *Papilio dardanus* (ABX27951), *Danaus plexippus* (OWR53873) and *D. melanogaster* (NP725057). The invected is homolog to engrailed, however, the results of phylogenetic analysis showed that the invected and engrailed were divided into two independent groups on the evolutionary tree, except for the invected of *D. melanogaster* was clustered with engrailed. 1000 bootstrap was used to evaluate the phylogenetic tree.
